# Scoliosis corrective force estimation from the implanted rod deformation using 3D-FEM analysis

**DOI:** 10.1186/1748-7161-10-S1-O23

**Published:** 2015-01-19

**Authors:** Yuichiro Abe, Manabu Ito, Kuniyoshi Abumi, Remel Salmingo, Shigeru Tadano, Hideki Sudo

**Affiliations:** 1 Eniwa Hospital, Eniwa, Japan; 2 Hokkaido University, Sapporo, Japan

## Introduction

Improvement of material property of spinal instrument has brought better deformity correction in scoliosis surgery in recent years. The increase of mechanical strength of instrument directly means the increase of force acts on bone-implant interface during scoliosis surgery. However, the actual correction force during correction maneuver and safety margin of pull out force on each screw were not well known. In the present study, estimated corrective forces and pullout forces were analyzed using a novel method based on Finite Element Analysis (FEA).

## Materials and methods

Twenty Adolescent idiopathic scoliosis patients (1 boy and 19 girls) who underwent reconstructive scoliosis surgery between June 2009 and Jun 2011 were included in this study. Scoliosis correction was performed with 6mm of diameter titanium rod (Ti6Al7Nb) using the simultaneous double rod rotation technique (SDRRT) in all cases. Material properties of this rod are Young's Modulus, yield stress, yield strain and hardening coefficient equal to 105 GPa, 900 MPa, 8.57x10-3 and 2.41 GPa, respectively. The pre-maneuver and post-maneuver rod geometry were collected from intraoperative tracing and postoperative 3D-CT images, and 3D-FEA was performed with ANSYS. Cobb angle of major curve, correction rate and thoracic kyphosis were measured on X-ray images.

## Results

Averaged age at surgery was 14.8 and averaged fusion length was 8.9 segments. Major curve was corrected from 63.1 to 18.1 degrees in average and correction rate was 71.4%. Rod geometry showed significant change on the concave side, and curvature of the rod on concave and convex side reduced from 33.6 to 17.8 degrees, from 25.9 to 23.8 degrees, respectively. Estimated pull-out forces at apical vertebrae were 160.0N in the concave side screw and 35.6N in the convex side screw. Estimated push-inn force at LIV and UIV were 305.1N in the concave side screw and 86.4N in the convex side screw.

## Discussion

Corrective force during scoliosis surgery was demonstrated to be greater in concave side about 4 times than in convex side. Averaged pull out and push in force fell below previously reported safety margin (400 to 600N/screw), therefore SDRRT maneuver was safe for correcting moderate magnitude curve. For prevent implant breakage or pedicle fracture during maneuver in severe curve correction, mobilization of spinal segment by releasing soft tissue or facet joint could be more important rather than the use of stronger correction maneuver with rigid implant.

